# Two chromatographic methods for determination of cytarabine and dexamethazone; two co-administered drugs for the treatment of leukemia: green analytical perspective

**DOI:** 10.1186/s13065-024-01184-5

**Published:** 2024-05-03

**Authors:** Maimana A. Magdy, Ahmed M. Elgebaly, Ahmed A. Taha, Nourddin W. Ali, Nehal F. Farid

**Affiliations:** 1 Pharmaceutical Analytical Chemistry Department, Faculty of Pharmacy, Beni-Suef University, Alshaheed Shehata Ahmad Hegazy St., Beni-Suef, 62511 Egypt; 2 Analytical Chemistry Department, Faculty of Pharmacy, Nahda University, Beni-Suef, Egypt; 3 Chemistry Department, Faculty for Girls, Ain Shams University, Cairo, Egypt

**Keywords:** Cytarabine HCl, Dexamethazone, Leukemia, HPLC, HPTLC, Human plasma

## Abstract

Two sensitive, straightforward and repeatable chromatographic techniques were developed for the determination of Cytarabine HCl and Dexamethazone in their pure form and spiked human plasma without prior separation. The drugs are used co-administered for the treatment of Leukemia, a certain type of blood cancer. Method (A) is an isocratic chromatographic HPLC method; separation was accomplished on C18 column using the eluting mixture of 6.9 g/L Monobasic Sodium Phosphate pH 3: methanol (70:30, v/v) and detection was at 275 nm. Concentrations were in the range of 0.2–15 μg/mL for both CYT and DEX. Method (B) is a HPTLC method in which separation was attained on HPTLC F254 plates using methanol: ethyl acetate: ammonia, (7.8:2:0.2, by volume) as eluting solvents and detection was at 275 nm. Concentrations were in the range of 0.1–4 μg/band for both CYT and DEX. The parameters for system suitability testing were evaluated to determine the effectiveness of the developed chromatographic procedures in terms of performance. The recently developed techniques were applied for the determination of the drugs under investigation in spiked human plasma. Validation parameters were examined in accordance with US-FDA criteria. All results were found to be within the acceptable ranges. To evaluate the greenness characters of the proposed methods to the environment; three greenness assessment tools including eco-scale assessments (ESA), green analytical procedure index (GAPI), and Analytical Greenness calculator (AGREE) were used. Acceptable and satisfying results that demonstrated the greenness characteristics of the suggested methods were attained.

## Introduction

Cytarabine HCl (CYT) and Dexamethasone (DEX) are co-prescribed in chemotherapeutic treatment of Acute Myeloid Leukemia [1]. The antimetabolite antineoplastic drug CYT prevents DNA synthesis. Its effects are particular for the S phase of the cell cycle. Additionally, it possesses immunosuppressant and antiviral activities. [2]. DEX, a corticosteroid, is used in the treatment of severe allergic reactions. DEX is also used to overcome drug resistance in T cell, so show chemosensitisation to the cytotoxic effects of chemotherapy on Acute Myeloid Leukemia cell lines [2].

**Fig. 1:**
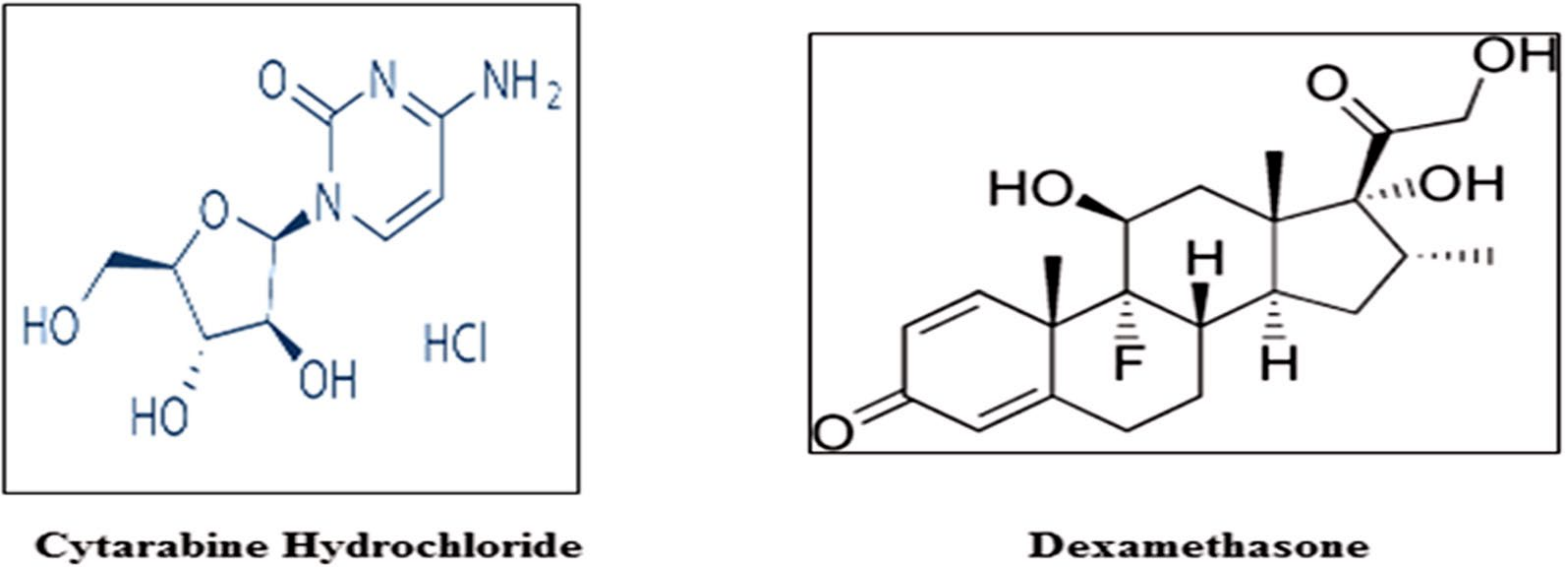
Chemical structures of Cytarabine Hydrochloride and Dexamethasone

CYT is chemically known as "4-amino-1-b-d-arabinofuranosylpyrimidin-2(1H)-one hydrochloride" Fig. 1**;** it is an official drug in both USP and BP [3, 4]. In literature variable methods were described for the estimation of Cytarabine HCl in drug products, either by itself or in mixture with other drugs [5–11].

DEX; 9-fluoro-glucocorticoid, Fig. 1, is official in USP, and BP [3, 4]. In literature; many methods for estimation of Dexamethasone in drug products, either by itself or in conjunction with other drugs have been published [12–16].

The binary mixture of CYT and DEX have been evaluated simultaneously by a UV-spectrophotometric method [17] in pure form, pharmaceutical formulations and biological fluids, solid phase extraction and chloroform were used for extraction of the drugs from biological fluids. Three greenness assessment tools including eco-scale assessments (ESA) [18], green analytical procedure index (GAPI)[19], and Analytical Greenness calculator (AGREE)[20], were used to measure and assess the greenness characters of the proposed methods and the previously published spectrophotometric one. Results reveal that both proposed chromatographic methods were found to be superior concerning greenness profile than the published spectrophotometric method [17] which used solid phase extraction utilizing chloroform as an extracting solvent. Chloroform is known to be a non-green hepatotoxic hazardous solvent. This work focuses on the development and validation of two chromatographic methods for determination of the proposed pharmaceuticals in their raw materials and in spiked human plasma. The suggested HPLC and HPTLC-densitometric methods, being chromatographic methods, have the advantage of being selective when compared to the published Spectrophotometric method [17].

## Experimental

### Instruments

A Thermo Scientific Dionex Ultimate S 3000 HPLC system with YMC-Pack HPLC column, C18 with dimensions of 15 cm × 2.1 mm, and particle size of 3 μm (Germany), high performance liquid chromatographic system was used for the separation of the studied drugs. Detector used was a Dionex ultimate 3000 RS diode array detector. An autosampler (Dionex Ultimate autosampler WPS-3000) was used for injection of samples.

HPTLC-densitometriy: HPTLC aluminum plates of 0.25 mm thickness (20 × 20) precoated with silica gel 60 F_254_ (Merck, Germany) were used; application was done using a 100 μL microsyringe 100 μL CAMAG Linomat 5, autosampler (Switzerland); development was achieved in glass tank (Macherey–Nagel, Germany). CAMAG HPTLC Densitometric Scanner 3S/N with WINCATS software (CAMAG, Switzerland) was used for scanning and measurement of bands.

### Materials

#### Pure samples

CYT was purchased from Egyptian International Medical Center (EIMC) United Pharmaceuticals Co. Cairo, Egypt, with a purity of 99.74 according to the company’s certificate. DEX was provided by Amriya for Pharmaceutical Industries, Alexandria, Egypt, with certified purity of 100.09% according to the official method [3].

### Internal standards

Alogliptin (AGT) was provided by Western Pharmaceutical Industries Co., with a purity of 100.07% according to the official method [3].

Diclofenac Na (DIC) was kindly provided by Medical Union Co. for Pharmaceuticals, Ismailia, Egypt. Its purity was 100.11% according to USP method [3].

#### Biological sample

The Aman Laboratory in Beni-suef, Egypt provided the blank human plasma samples, which were drawn from six healthy people.

#### Chemicals and reagents

Methanol HPLC grade (CHROMASOLVE®, Sigma -Aldrich Chemie GmbH, Germany) was used. Monobasic Sodium Phosphate was obtained from Loba Chemie Pvt Ltd, Mumbai, India. Analytical grade solvents and reagents used including ethyl acetate, phosphoric acid and ammonia were purchased from El-Nasr Pharmaceutical Chemicals Co., Cairo, Egypt. Deionized water was obtained from SEDICO Pharmaceutical Co., 6th of October City, Egypt.

### Solutions

For HPLC and HPTLC: Stock standard solutions of 1 mg/mL in methanol were prepared for each of CYT, DEX, AGT and DIC (both AGT and DIC were used as internal standards), where 25 mg of each component was weighed in separate 25 mL volumetric flask, volumes were adjusted using methanol.

Working standard solutions were prepared by separately transferring 2.5 mL of each drug from their corresponding stock standard solutions (1 mg/mL) into four 25 mL volumetric flasks, the volume of each solution was completed with methanol to prepare a working solution of 0.1 mg/mL.

## Procedure

### Calibration curves and regression equations

*For HPLC:* Different aliquots of CYT and DEX in the ranges of 2–150 µg were taken from their respective working solutions into two sequences of 10-mL volumetric flasks, to each flask 0.7 mL of the working solution of AGT was added and the volume of each flask was adjusted with water: methanol (70:30, v/v). Concentrations were in the range of 0.2–15 μg/mL for both CYT and DEX and was 7 μg/mL for AGT (internal standard). A volume of 20 µL from each flask was injected and resolution of the components was achieved within 12 min using C18 column through which an eluent was forced at a flow rate of 1 mL/mim. The eluent consisted of 6.9 g/L monobasic sodium phosphate, pH 3, adjusted by ortho phosphoric acid: methanol in the ratio 70:30, v/v. The detector was set at 275nm. Peak area measurements were taken, and integrated peak area ratios; peak areas of each analyte divided by the peak areas of the internal standard (AGT) were calculated and utilized to build calibration curves. Then, regression equations were computed for pure samples and found to be:

$${{\text{Y}}}_{{\text{CYT}}} = 5.1513{{\text{C}}}_{{\text{CYT}}} + 13.031 \, \mathrm{ r }= 0.9999\, \mathrm{ for}\, CYT$$

$${{\text{Y}}}_{{\text{DEX}}}=3.9874 {{\text{C}}}_{{\text{DEX}}} + 5.5971 \, \mathrm{ r }= 0.9999\, \mathrm{ for} \,DEX$$

where Y is the peak area ratio (Analyte/I.S), C is the concentration in µg/ mL and r is the correlation coefficient.

*For HPTLC:* Different CYT and DEX concentrations, each ranging from 0.1–4 mg, were delivered separately into two sets of 10-mL measuring flasks. Each flask received 2 mg of DIC as an internal standard. The flasks were shaken before being completed with methanol to the mark. Triplicates application of 10- μL from each flask in the form of bands of 6 mm width were then applied to HPTLC plates. Bands were 8.9 mm apart and were applied to the plates using a microsyringe at a speed of 20 mm/s, the slit size was set to 6.0 × 0.3 μm. An eluting system consisting of methanol: ethyl acetate: ammonia, (7.8:2:0.2, by volume) was used. The chromatographic development glass unit was allowed to be saturated with the eluent for 15 min after which the plate was placed and left till the eluent reached 8 cm. Spectrophotometric scanning was conducted at 275 nm. Peak areas were measured, and integrated peak area ratios; peak areas of each analyte divided by peak areas of the internal standard employed (DIC); were calculated and plotted against concentration for the generation of calibration curves; from which regression equations were then computed and found to be:

$${{\text{Y}}}_{{\text{CYT}}}=-0.0355{{{\text{C}}}^{2}}_{{\text{CYT}}} +1.0993{{\text{C}}}_{{\text{CYT}}}+ 0.077\, \mathrm{ r }= 0.9996\, \mathrm{ for}\, CYT$$

$${{\text{Y}}}_{{\text{DEX}}}= -0.1554 {{{\text{C}}}^{2}}_{{\text{DEX}}}+ 1.8121{{\text{C}}}_{{\text{DEX}}} +0.29 \, \mathrm{ r }= 0.9993\, \mathrm{ for}\, DEX$$

where Y is the peak area ratio (Analyte/I.S), C is the concentration in µg/ band and r is the correlation coefficient.

### Spiked human plasma

*For HPLC method:* Samples of CYT and DEX were transferred independently into two separate series of 10 mL measuring flasks in the range of 2–150 g from their previously prepared working solutions, 0.7 mL of AGT as internal standard (0.1 mg/mL) was added followed by 1 mL plasma. Plasma protein was precipitated by the addition of 2 mL methanol and the volumes of the flasks were adjusted using water: methanol (70:30, v/v).

Samples were vortexed for 1 min then centrifuged at 5000 rpm for 5 min in a cooling centrifuge to remove the precipitated plasma protein. Afterwards, samples were filtered using 0.45 μm, Acrodisc syringe filter. Steps have been taken in accordance with linearity, peak areas were then measured for the studied drugs and the internal standard. The integrated peak area ratios [peak area of the each of the two analytes/ peak area of AGT internal standard] were computed and used together with concentrations for generation of the calibration curves. Regression equations were then calculated and found to be:

$${{\text{Y}}}_{{\text{CYT}}}=5.0103{{\text{C}}}_{{\text{CYT}}}+ 12.570 \, \mathrm{ r }= 0.9998\, \mathrm{ for} \, CYT$$

$${{\text{Y}}}_{{\text{DEX}}}= 3.1955{{\text{C}}}_{{\text{DEX}}} + 5.4398 \, \mathrm{ r }= 0.9998 \, \mathrm{ for}\, DEX$$

where Y is the peak area ratio (Analyte/I.S), C is the concentration in µg/ mL and r is the correlation coefficient.

*For HPTLC method:* Different amounts of CYT and DEX samples were placed in two independent groups of 10 mL volumetric flasks in the ranges of 0.1–4 mg, 2 mg of DIC (used as an internal standard) was then added to each flask followed by 1 mL plasma, and the volume was finished with methanol. Solutions were then mixed well and vortexed for one minute, then plasma protein was removed by placing all the solutions in a cooling centrifuge for 5min at 5000ppm. Samples were then filtered through 0.45 μm Ac rodisc filter (PN MS-320I) and 10 μL were applied in triplicates on HPTLC plates then procedures under linearity was followed. Peak area ratios (area under the peak of analyte/ area under the peak of DIC) were recorded then regression equations have been computed and found to be:

$${{\text{Y}}}_{{\text{CYT}}}=-0.0356{{{\text{C}}}^{2}}_{{\text{CYT}}} +1.1028{{\text{C}}}_{{\text{CYT}}}+ 0.072\, \mathrm{ r }= 0.9994\, \mathrm{ for}\, CYT$$

$${{\text{Y}}}_{{\text{DEX}}}= -0.1564{{{\text{C}}}^{2}}_{{\text{DEX}}}+1.8171{{\text{C}}}_{{\text{DEX}}} +0.2898\, \mathrm{ r }= 0.9997\, \mathrm{ for}\, DEX$$

where Y is the peak area ratio (Analyte/I.S), C is the concentration in µg/ band and r is the correlation coefficient.

Several concentrations were chosen to prepare quality control samples which were prepared in a similar way as calibration samples; chosen concentrations were 0.2 µg/mL (LLOQ), 3 µg/mL (LQC), 8 µg/mL (MQC) and 15 µg/mL (HQC) for both CYT and DEX for HPLC method and concentrations of 0.1 µg/band (LLOQ), 1 µg/band (LQC), 2 µg/band (MQC) and 4 µg/band (HQC) for both CYT and DEX for HPTLC method.

This study was approved by The Research Ethical Committee of Faculty of Pharmacy, Beni-Suef University, (Serial No.: REC-H-PhBSU-24007).

## Results and discussion

In the regimen for treating leukaemia, especially acute non-lymphoblastic leukaemia, CYT and DEX are used. The two drugs have two different pharmaceutical formulations and are co-prescribed in the treatment of Leukemia. DNA synthesis is inhibited by the antimetabolite, antineoplastic drug CYT. Its actions are specific for the S phase of the cell cycle, using DEX, overcome drug resistance in T cell, so show chemosensitisation to the cytotoxic effects of chemotherapy on acute myeloid leukaemias cell lines [1].

Trials were conducted in this work to develop HPLC and HPTLC methods that could separate and quantify CYT and DEX in a short period of time with a high level of sensitivity and selectivity. Additionally, attempts were made to use less dangerous solvents. According to their negative impacts on the environment, organic solvents were categorized into three groups: desirable, acceptable, and undesirable. To employ desirable solvents, numerous tests were conducted.

### Method development and optimization

#### HPLC method

**Fig. 2:**
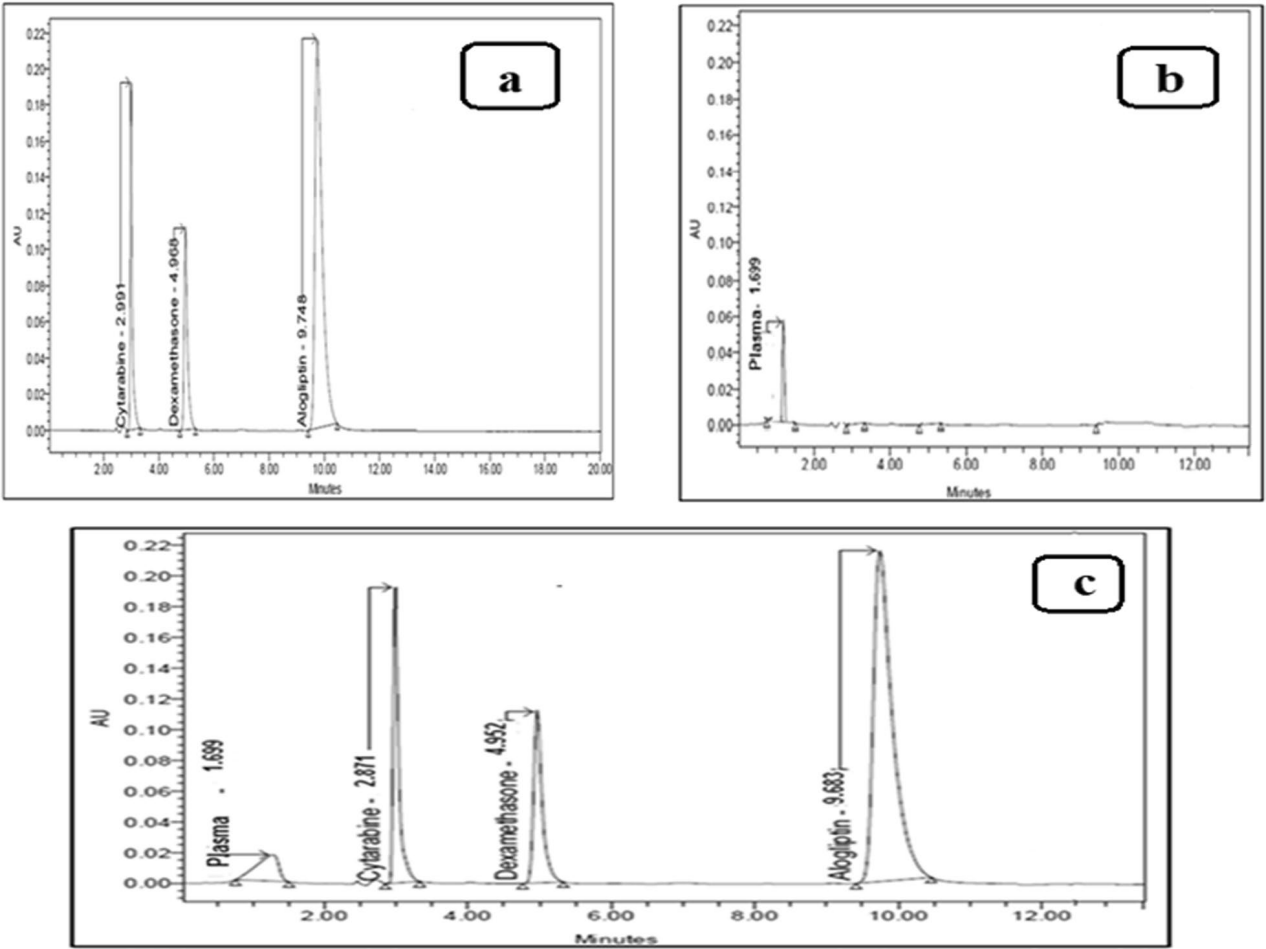
**a** RP-HPLC chromatogram of mixture of Cytarabine HCl 4 µg mL^−1^ and Dexamethasone 2 µg mL^−1^, in presence of 7 µg mL^−1^ Alogliptin benzoate as internal standard, using a mobile phase of 6.9 g/L Monobasic Sodium Phosphate: methanol (70:30, v:v), **b** RP-HPLC chromatogram of plasma, **c** RP-HPLC chromatogram of mixture of Cytarabine HCl 4 µg mL^−1^ and Dexamethasone 2 µg mL^−1^, in presence of 7 µg mL^−1^ Alogliptin benzoate as internal standard, in spiked human plasma

Different mobile phases were tried using acetonitrile, methanol as organic modifiers and using water as aqueous solvent (50:50, v/v), flow rate was initially set at one mL/min, detector was adjusted at 254 nm, and C8 column as a stationary phase was used. Good separation was achieved upon using methanol as organic modifier and water as aqueous solvent but with forked peak for CYT and tailed peak for DEX. Afterwards, the amount of water was raised (up to 70%), regrettably, poor separation among the studied drugs was found. Additional tests were done by altering the aqueous phase pH, in the range of 3–9 by the use of ortho phosphoric acid, glacial acetic acid or mono basic sodium phosphate, however, no improvement was observed. The stationary phase was then replaced with reversed phase C18 column, which resulted in great improvement in resolution and peak shape. The ratio (30:70, v/v), methanol: 6.9 g/L monobasic sodium phosphate resulted in complete resolution among the peaks of the drugs under investigation and plasma peak with suitable analysis time. Different detecting wavelengths were examined to enhance sensitivity (220, 254 and 275 nm). The wavelength 275 nm was adequate for detection of CYT and DEX. Several internal standards were tried and AGT was found to be suitable with respect to retention time and resolution from the studied drugs, Fig. 2.

#### HPTLC method

To acquire the best resolution and peak symmetry, tests were conducted to determine the optimal mobile phase. Ethyl acetate and methanol were used to achieve a decent separation, although DEX showed a tailed peak. The use of ammonia resulted in appreciable improvement. The used eluent was methanol: ethyl acetate: ammonia, (7.8:2:0.2, by volume). It was discovered that a 15-min saturation period was suitable for effective resolution. Various scanning wavelengths were assessed (225, 254 and 275 nm). High base line noise was produced by detection at 225 nm, whereas lesser sensitivity was produced at 254 nm. The optimal signal to noise ratio for the two components was obtained with detection at 275 nm. Plasma peaks were almost retained at the base line of the stationary phase and therefore did not affect separation of the studied drugs.

**Fig. 3:**
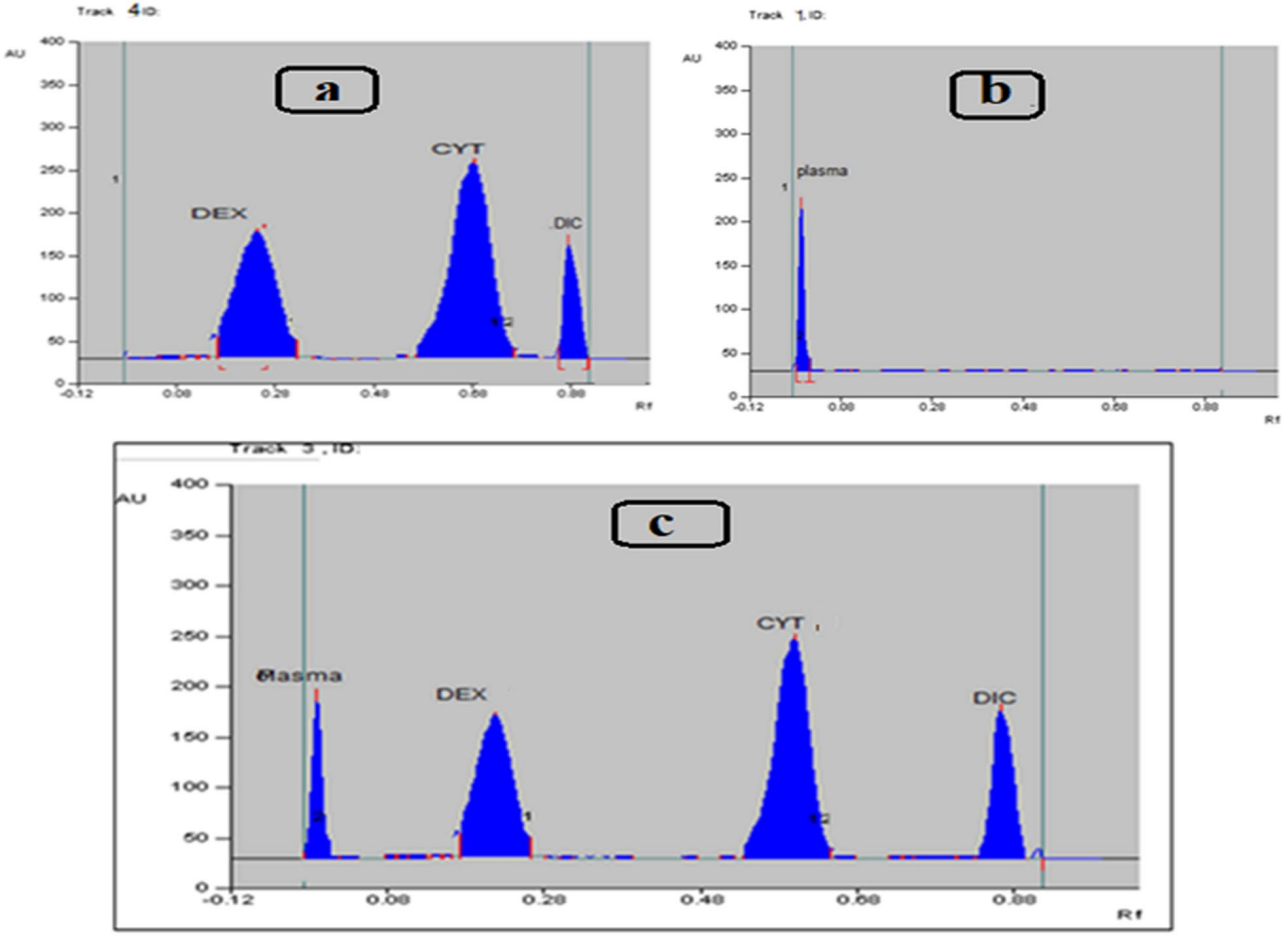
**a** 2D TLC Densitogram of mixture of Dexamethasone and Cytarabine HCl using methanol: ethyl acetate: ammonia, (7.8:2:0.2 v/v/v) as a developing system at 275 nm in presence of Diclofenac Na as an internal standard. **b** TLC Densitogram of plasma. **c** 2D TLC Densitogram of mixture of Dexamethasone and Cytarabine HCl in presence of Diclofenac Na as an internal standard, in spiked human plasma

**Fig. 4:**
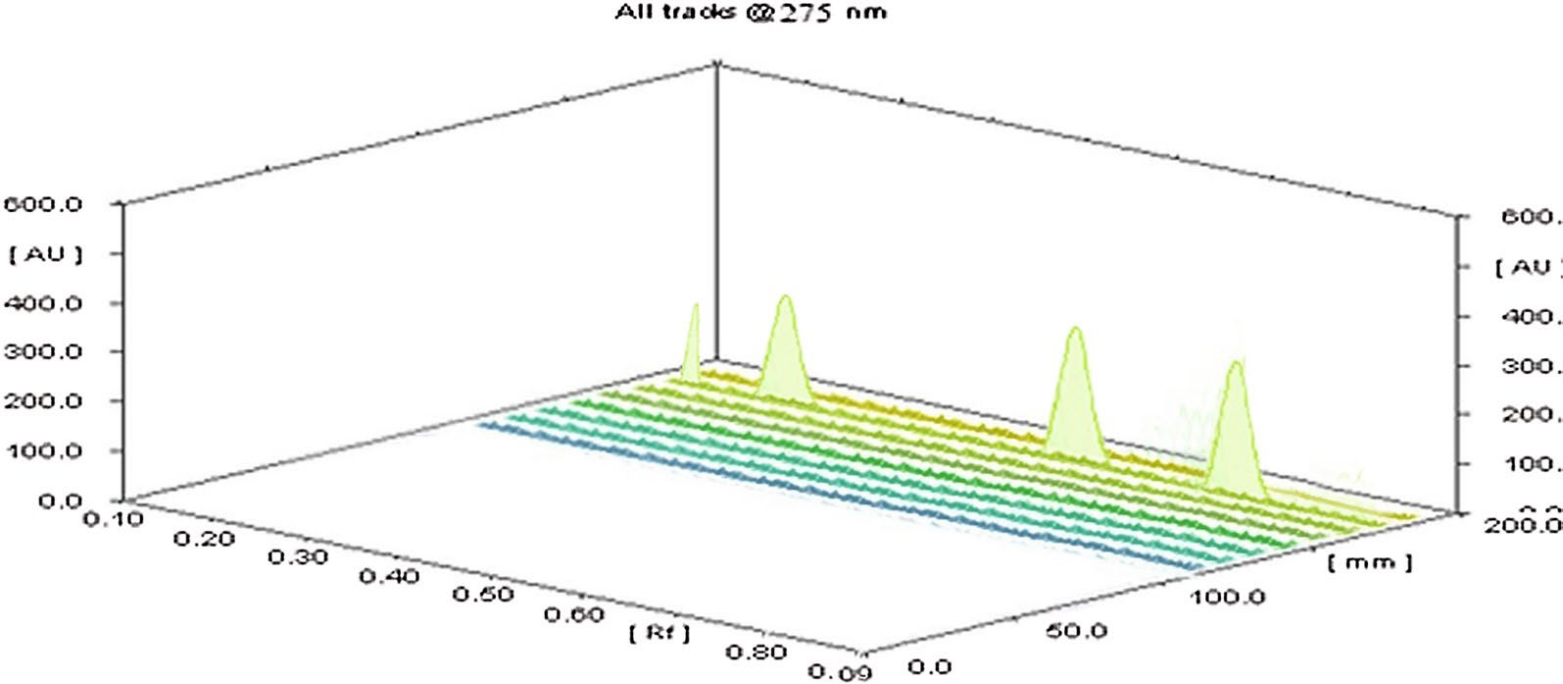
3D TLC Densitogram of mixture of Dexamethasone and Cytarabine HCl using Diclofenac Na as an internal standard in spiked human plasma

Considering the variability of analyte loss during sample treatment, internal standards must be utilized when creating bioanalytical procedures. Several internal standards were tried, the most suitable ones concerning the chromatographic characteristics and resolution were AGT benzoate and DIC for HPLC and HPTLC, respectively Figs. 3 and 4.

### Method validation

#### Analytical method validation

The method validation stage has been performed in accordance with USP [3] guidelines. Following evaluations of linearity, accuracy, precision, LOD, and LOQ, the findings are presented in Table 1.

**Table 1:** Regression and validation parameters for the analysis of Cytarabine HCl and Dexamethasone in pure samples and in spiked human plasma by the proposed chromatographic methods

Parameters	Pure samples			
	HPLC		HPTLC	
	CYT (μg/ mL)	DEX (μg/ mL)	CYT (μg/band)	DEX (μg/band)
Range^a^	0.2–15	0.2–15	0.1–4	0.1–4
Slope	5.1513	3.9874	− 0.0355 1.0993	− 0.1554 1.8121
Intercept	13.034	5.5971	0.077	0.29
Correlation (r)	0.9999	0.9999	0.9996	0.9993
Accuracy^b^	99.31	99.74	99.20	99.90
Repeatability^c^	0.934	0.808	1.44	1.47
Intermediate precision (%RSD)^c^	1.27	0.990	1.76	1.84
*Robustness (%RSD)*				
- Eluent flow rate (± 0.05 mL/min) - Methanol (70 ± 1%) - Ethyl acetate (2 ± 1%) - Ammonia (0.2 ± 0.01 mL) - wavelength (± 2 nm)	1.73 2.41 – – 0.33	1.12 1.84 – – 0.79	– – 0.74 0.02 0.82	– – 0.41 0.02 0.79
Ruggedness (Different methanol manufacturers)	0.77	0.62		
LOD^d^	0.051	0.048	0.024	0.027
LOQ^d^	0.17	0.16	0.08	0.09

Parameters	Plasma samples			
	HPLC		HPTLC	
	CYT (μg/ mL)	DEX (μg/ mL)	CYT (μg/band)	DEX (μg/band)
Range^a^	0.2–15	0.2–15	0.1–4	0.1–4
Slope	5.0103	3.955	− 0.0356 1.1028	− 0.1564 1.8171
Intercept	12.570	5.4398	0.0722	0.2898
Correlation (r)	0.9998	0.9998	0.9994	0.9997
LLOQ	0.2	0.2	0.1	0.1
ULOQ	15	15	4	4

^a^ Calibration was attained using the linear regression equation in case of HPLC and polynomial regression equation: A = aX2 + bX + C in case of HPTLC, A = peak area ratio, X is the concentration and C is the intercept

^b^ mean of 8 concentrations for each drug ^c^ Intra- and inter-day RSD of three concentrations of each component, 3, 6 and 12 μg/ mL for HPLC method, and 1, 2 and 4 μg/band for HPTLC

^d^ LOD = (3.3 X SD)/slope (SD of the intercept using the lower part of the calibration graph, the slope of the calibration curve; LOQ = (10X SD)/slope

Drugs under investigation were completely separated from one another and from the plasma peak under the used chromatographic settings, demonstrating the method's selectivity, moreover, no additional interfering peaks were seen in the chromatograms, as illustrated in Figs. 2, 3, indicating that there was no influence from endogenous substances in the plasma matrices.

Robustness was investigated, and all the results were acceptable, demonstrating that the suggested procedures were unaffected by the minor changes made to the investigated parameters. The experimental conditions were deliberately changed whereas composition of each component of the developing system of the proposed methods was changed by ± 1%, while the wavelength was changed by (± 2 nm). In HPLC method; eluent flow rate was changed by ± 0.05 mL/min. Good results of %RSD were obtained as shown in Table 1.

#### System suitability testing parameters

Different chromatographic parameters were determined to govern the system appropriateness. Table 2 findings revealed that selectivity and resolution factor values fall within acknowledged limits, indicating successful chromatographic separation.

**Table 2:** System suitability testing parameters of the proposed chromatographic methods

Parameters	HPLC				HPTLC			
	Plasma	CYT	DEX	AGT	Plasma	DEX	CYT	DIC
Rt (for HPLC) or Rf (for HPTLC)	1.7	2.87	4.95	9.68	0.06	0.25	0.71	0.85
Peak symmetry	_	1.25	1.33	1.29	–	0.9	0.91	1.00
Selectivity (α)	2.56	2.08		2.18	5.22	4.69		3.55
Resolution (Rs)	2.72	4.18		5.86	3.73	4.12		3.43
Capacity factor (K’)	–	2.02	4.21	9.19	–	3	0.64	0.18
Number of theoretical plates (N)	–	1024	1764	1529	–	–	–	–
Height Equivalent to theoretical plate (H) in cm		0.015	0.0085	0.01				

Results obtained by applying the proposed methods were statistically compared with those obtained by applying the reported spectrophotometric method [17] for determination of the proposed drugs in their pure forms and no significance differences were obtained between them as shown in Table 3. The test ascertains that the proposed methods are as precise and accurate as the reported spectrophotometric method [17]

**Table 3:** Statistical comparison of the proposed Chromatographic methods and the reported methods for determination of Cytarabine HCl and Dexamethasone in their pure form

	TLC		HPLC		Reported method [17]*	
	CYT	DEX	CYT	DEX	CYT	DEX
Mean	99.20	99.91	99.31	99.74	100.17	100.23
SD	1.271	1.727	1.278	1.279	1.69	1.75
n	8	7	8	9	9	9
Variance	1.616	2.98	1.632	1.631	2.87	3.06
t-test	1.35 (2.13)**	0.372 (2.16)**	1.20 (2.13)**	0.686 (2.13)**		
f-value	1.768 (3.72)**	1.027 (4.14)**	1.760 (3.72)**	1.869 (3.43)**		

^*^Reported method [17]*First- and Third-Derivative Spectrophotometry for Simultaneous Determination of Dexamethasone and Cytarabine, in the zero-crossing wavelengths at 268.0 nm (first derivative) and 264.0 nm (third derivative) for determining dexamethasone and cytarabine, respectively

^**^ Figures between parentheses represent the corresponding tabulated values of *t* and *F* at *p* = 0.05

#### Bio‑analytical method validation

The FDA [21] standards for validating bio-analytical methods were followed. The evaluations of linearity, accuracy, precision, and selectivity are given in Table 1. Plasma samples spiked with CYT or DEX were used to create calibration curves for both pharmaceuticals by HPLC and HPTLC, in the concentration ranges of 0.2–15.0 µg/mL and 0.1–4 µg/band, respectively. Peak area ratios and linear regression was used in case of HPLC, while for HPTLC polynomial regression was used. All the resulting correlation coefficients were more than 0.9994. All regression factors are given in Table 1.

The four quality control samples (LLOQ, LQC, MQC, and HQC) were used to determine the accuracy of the proposed methods and were expressed as percentage recovery and bias%. All values with the exception of LLOQ should be 100 ± 15%, according to FDA-criteria [21], LLOQ can be 100 ± 20% of the true value. Results given in Table 4 indicate that all values were within the acceptable limit suggested by FDA for the two proposed chromatographic methods. Moreover the precision of both methods were evaluated, where repeatability and intermediate precision were assessed by analyzing the three quality control samples together with LLOQ, five times each and results were expressed as relative standard deviation (%RSD). According to findings in Table 4 all values fell within the acceptance range which were less or equal to 15% (for LQC, MQC and HQC) and 20% for LLOQ.

**Table 4:** Intra and inter assay precision and accuracy in plasma samples

	Conc(μg/mL)^a^	Intraday			Interday		
		Recovery %	Bias %^b^	RSD%	Recovery%	Bias %^b^	RSD%
*HPLC method*							
CYT	0.2(LLOQ)	104.67	4.67	6.71	103.85	3.85	7.59
	3(LQC)	105.20	5.20	3.34	106.57	6.57	4.42
	8(MQC)	103.60	3.60	3.18	104.63	4.63	4.31
	15(HQC)	104.46	4.46	5.07	104.69	4.69	5.61
DEX	0.2(LLOQ)	94.31	4.69	5.14	95.30	4.70	5.91
	3(LQC)	96.52	3.48	2.12	95.39	4.61	2.43
	8(MQC)	97.10	2.90	2.22	96.85	3.15	2.74
	15(HQC)	97.51	2.49	3.05	94.84	5.16	3.92
*HPTLC method*							
CYT	0.1(LLOQ)	102.05	2.05	3.91	109.41	9.41	7.42
	1(LQC)	97.88	2.12	3.01	101.87	1.87	4.11
	2(MQC)	98.17	1.83	2.96	98.33	1.67	3.91
	4(HQC)	98.49	1.51	3.26	102.47	2.47	4.52
DEX	0.1(LLOQ)	97.34	2.66	3.77	96.41	3.59	7.11
	1(LQC)	96.58	3.42	2.91	96.48	3.52	4.13
	2(MQC)	97.98	2.02	2.71	97.94	2.06	3.88
	4(HQC)	95.98	4.02	3.04	95.71	4.29	4.43

^a^ Mean of 5 experiments ^b^ % of deviation from true value

##### Recovery

Recovery of both methods were tested by analyzing three extracted samples at each quality control level (LQC, MQC and HQC) and comparing them to the recovery of three extracted blank plasma spiked with the same drug concentration after extraction.

Recovery percentage was calculated by dividing the area of extracted plasma samples by the area of blank plasma spiked with analyte post extraction and multiplying by 100, results are shown in Table 5.

**Table 5:** Extraction recovery of cytarabine HCl and dexamethasone in spiked human plasma

CYT		DEX	
Conc(μg/mL)^a^	Recovery%	Conc(μg/mL)^a^	Recovery%
*HPLC method*			
3	101.21	3	98.45
8	98.54	8	99.97
15	100.71	15	100.21
Mean ± SD	100.27 ± 1.56	Mean ± SD	99.68 ± 0.715
*HPTLC method*			
1	98.13	1	98.08
2	100.32	2	99.86
4	101.87	4	101.95
Mean ± SD	100.01 ± 2.03	Mean ± SD	99.95 ± 1.95

^a^ Mean of 3 determinations

###### Freeze and thaw cycle stability

Quality control samples (LOQ, MQC and HQC) for each drug by both methods were stored at − 20 °C and exposed to three freeze–thaw cycles. Results in Table 6 confirm the stability of the samples; as an alteration of less than 15% of the analyte concentration was obtained.

**Table 6:** Results of freezing–thawing and short term stability study for Cytarabine HCl and Dexamethasone in spiked human plasma samples

	Concentration (µg /band)	Recovery % ^a^	
		Three freeze thaw cycles	Bench top stability
	*TLC method*		
CYT	1	104.06	102.58
	2	107.29	104.11
	4	104.32	104.49
Mean ± SD		105.22 ± 1.79	103.72 ± 1.01
DEX	1	103.72	102.72
	2	106.96	104.96
	4	104.32	105.65
Mean ± SD		105.00 ± 1.72	104.44 ± 1.53
	*HPLC method*		
CYT	3	106.53	101.61
	8	106.88	102.55
	15	104.8	102.33
Mean ± SD		106.07 ± 1.11	102.16 ± 0.49
DEX	3	107.53	102.61
	8	106.21	103.35
	15	106.05	102.5
Mean ± SD		106.59 ± 0.81	102.82 ± 0.46

^a^ Average of 3 determinations

##### Short term temperature stability

Quality control samples were left for 24 h at room temperature then analyzed and percentage relative standard deviation was calculated. Results were summarized in Table 6, which prove that all analytes were stable under normal working conditions.

### Greenness assessment of the HPLC and HPTLC method

Different analytical parameters and methods using green analytical chemistry (GAC) tools were used to look for less eco-friendly components that could be replaced by more green ones to meet GAC standards. There are now several criteria for determining how ecologically sustainable analytical processes are. Several metrics were developed to evaluate the greenness of an analytical method, each tool has some benefits and drawbacks.

Making the final choice could be difficult because the outcomes of each tool may generate different suggestions for the optimal greenness technique to apply. So it is better and beneficial to use a variety of tools.

#### Eco-scale assessment (ESA)

The analytical Eco-scale [18] is one of the recognized metrics for evaluating the method's environmental friendliness. Results in Table 7, show that the proposed HPLC method has a score of 83, and a score of 80 for the proposed HPTLC method, compared to the published spectrophotometric method [17] which has a score of 82; The method is considered to be absolutely environmentally friendly if its score is 100, outstanding if it is 75 or higher, reasonable if it is 50 or higher, and insufficient if it is lower than 50.

**Table 7:**
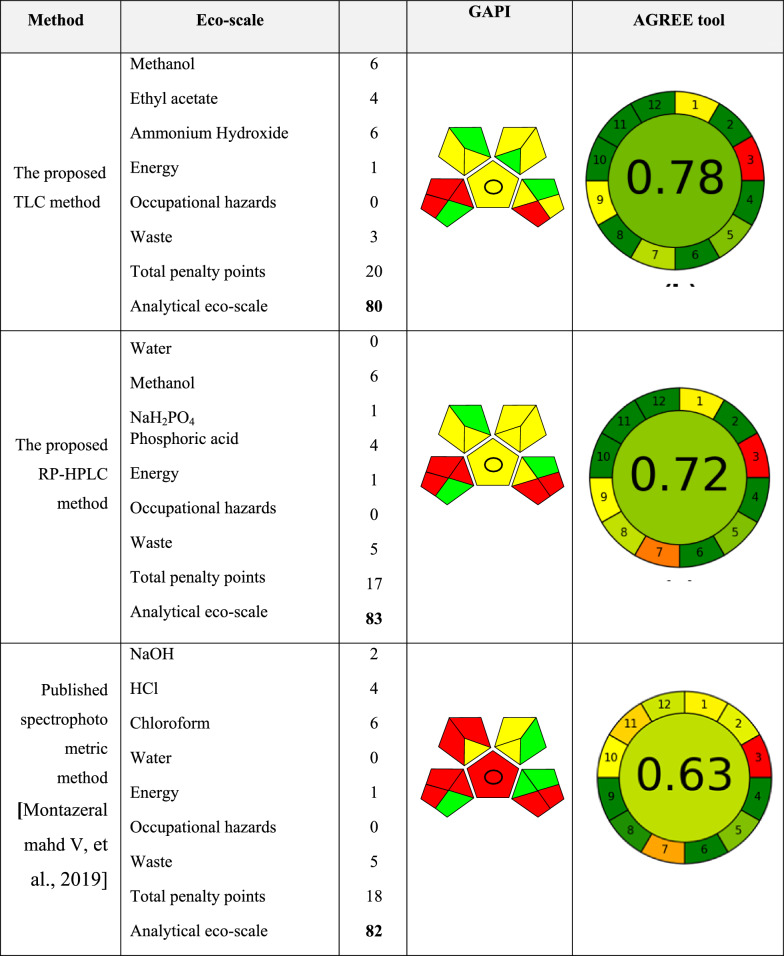
The greenness profile of the proposed chromatographic methods using), analytical eco-scale, GAPI and Agree tools

#### Green analytical procedure index (GAPI)

The GAPI [19] pictogram for the proposed methods and the reported spectrophotometric method [17] are presented in Table 7. The GAPI system of a variety of factors, including reagents, apparatus, and sample preparation were applied. Four green, seven yellow, and four red sections were produced in HPTLC method, while in HPLC method, three green, seven yellow, and five red sections were produced when examining the GAPI pictograms in Table 7, one can see that the reported spectrophotometric method has the greatest proportion of red fields (eight red sections) this is due to the use of toxic non-green solvent (chloroform) and special steps required for sample preparation (solid phase extraction).

#### Analytical greenness calculator (AGREE)

AGREE, can be thought of as a fast quantitative tool, employing freely available and simple software, which produces a score indicating how well a method adheres with the twelve guiding principles of green analytical chemistry [20]. Higher scores indicate greener approaches, with the complete score being presented in the middle of a round pictogram. The newly developed HPTLC received the highest score (0.7), according to Table 7, when compared to the previously published spectrophotometric method (0.63) [17] and the newly developed HPLC method (score 0.66].

According to Table 7, comparing the green assessment of the proposed methods with the published spectrophotometric one using different tools showed that the proposed ones have less hazards on the environment.

## Conclusion

This research work presents HPLC and TLC procedures that are robust, sensitive, selective and accurate for the resolution and measurement of CYT and DEX in spiked human plasma. The effectiveness of the two analytical approaches for the analysis of CYT and DEX was assessed under optimum experimental conditions. All factors for validation fulfilled the FDA acceptance criteria. The findings of evaluating and comparing the two proposed procedures together with the reported spectrophotometric method using three green assessment tools showed that the suggested chromatographic methods were comparatively ecologically benign and had few dangers.

## Data Availability

Availability of data and materials: the datasets used and/or analysed during the current study available from the corresponding author on reasonable request.

## Abbreviations

CYT: Cytarabine HCl
DEX: Dexamethasone
